# Assessing the Value of Recreational Divers for Censusing Elasmobranchs

**DOI:** 10.1371/journal.pone.0025609

**Published:** 2011-10-10

**Authors:** Christine A. Ward-Paige, Heike K. Lotze

**Affiliations:** Department of Biology, Dalhousie University, Halifax, Canada; University of Pretoria, South Africa

## Abstract

**Background:**

Around the world, researchers are using the observations and experiences of citizens to describe patterns in animal populations. This data is often collected via ongoing sampling or by synthesizing past experiences. Since elasmobranchs are relatively rare, obtaining data for broad-scale trend analysis requires high sampling effort. Elasmobranchs are also relatively large and conspicuous and therefore it may be possible to enlist recreational divers to collect data on their occurrence and relative abundance from daily dive activities. For this, however, a good understanding of the value of data collected by recreational divers is essential.

**Methodology/Principal Findings:**

Here, we explore the value of recreational divers for censusing elasmobranchs using a diverse set of data sources. First, we use a simulation experiment to explore detection rates of the roving diver technique, used by recreational divers, across a range of fish densities and speeds. Next, using a field survey, we show that inexperienced recreational divers detect and count elasmobranchs as well as experienced recreational divers. Finally, we use semi-structured interviews of recreational dive instructors to demonstrate the value of their recollections in terms of effort and their descriptions of spatial and temporal distributions of sharks in Thailand.

**Conclusions/Significance:**

Overall, this study provides initial ground-work for using recreational divers for monitoring elasmobranch populations. If used appropriately, citizen-collected data may provide additional information that can be used to complement more standardized surveys and to describe population trends across a range of spatial and temporal scales. Due to the non-extractive nature of this data, recreational divers may also provide important insight into the success of conservation initiatives, such as shark sanctuaries and no-take zones.

## Introduction

Scientists have been gathering data based on the experiences of citizen observers (e.g. citizen scientists and resource users) to describe patterns in animal populations for more than a century [1]–[7]. Because elasmobranchs are highly mobile, widely distributed, relatively rare fishes with large home ranges it is often not logistically or economically feasible for scientists to conduct visual censuses for broad-scale trend analysis. However, since elasmobranchs are also largely conspicuous species that inhabit a wide range of depths, temperatures, and habitats, it may be possible to enlist professional and recreational scuba divers, with a wide range of interests, to collect and report valuable data on their occurrence and abundance. Citizen-based programs with state-of-the-art survey design and data analysis can provide relatively reliable data with unbiased results [4], even with very little observer training [8], [9]. General trends in fish populations [10]–[12], including a few that comprise elasmobranchs [13]–[17], have been generated from data collected by citizen divers (i.e. recreational divers). However, all these projects used trained divers, which has advantages but also limits the number of participants and therefore areas and years sampled. In the present study, we explore the value of using any recreational diver for describing broad patterns in elasmobranch populations.

To effectively use diver observations for elasmobranch censuses it would be ideal to maximize sampling effort to allow for longer, more broad-scale and detailed descriptions. Today, PADI (www.padi.com)–the world's largest recreational diving membership organization–awards >900,000 certifications (>300,000 beyond entry level) per year and has >130,000 worldwide registered professional members (Divemaster or higher) (www.padi.com/scuba/about-padi/PADI-statistics/default.aspx). Thus, although there may be a significant dropout, based on the sheer number of divers worldwide combined with a growing appreciation of elasmobranchs [18], [19], recreational diver observations may be a viable source of data.

Scientists have been using underwater visual censuses (UVC) since the 1950's to census fish communities [20]. Although a few studies have included elasmobranchs [21]–[24], they are often overlooked or excluded where they occur at low abundance because they rarely enter the survey boundaries [25]. Typical scientific UVC limit fish counts within delineated boundaries (e.g. belt-transect and stationary point count, [20], [26]), whereas recreational divers move around a dive site, visually scanning the water column – often moving towards objects of interest. This type of roving dive [27] has the added benefit of detecting fish anywhere in the water column, in any habitat and at any time during the dive. Although survey boundaries are not defined and fish length is not measured during a roving dive as they often are in scientific dives, which excludes estimates of absolute density and biomass, occupancy and relative abundance measures are invaluable [14]–[16] given the sparse data [28]. Also, because roving divers survey larger areas than most scientific methods, the chance of detecting rare fish is increased.

Recreational divers tend to visit the best available sites on a regular basis. The ‘best site’ is subjective, but for many divers it includes charismatic megafauna, like sharks [19], [29]. Divers' experiences at these sites, if collected and analyzed appropriately, are currently an under-utilized source of data. Although a number of distractions (e.g. gear, buddy, buoyancy control) can inhibit a diver from accurately observing their surroundings, as a diver becomes more experienced these distractions are minimized and corresponding observations should be more accurate. Experienced recreational divers (e.g. recreational dive instructors) often become so familiar with the features of regularly visited sites that they can vividly describe the location of many stationary fishes (e.g. clown fish) and can provide directions to the exact crevices where highly mobile cryptic species, such as wobbegong sharks, can be found (CWP personal observation).

Citizen experiences are typically collected in two ways. The first deploys resource users as citizen scientists [2] to report their individual observations. This practice often depends on interested, semi-trained to expert observers using specified techniques that visit particular sites at specified times of the day and year (e.g. Christmas Bird Count, birds.audubon.org/Christmas-Bird-Count; Breeding Bird Survey, www.pwrc.usgs.gom/BBS/). Other projects are more flexible and include observations made at any time of the day or year such as the Reef Environmental Education Foundation (REEF, www.reef.org). However, in 2002 the Cornell Lab of Ornithology and the National Audubon Society launched one of the most adaptable citizen science programs. Project eBird (www.ebird.org) engages everyday birders (trained or not) to report their bird observations using a range of sampling protocols at any time of the day or year. Since its release, eBird has collected more than 21 million bird records from over 35,000 unique observers on 180,000 locations, thus creating a near real-time resource [3].

The second way citizen experiences are collated is through structured or semi-structured surveys and interviews that summarize individuals past effort and observations (e.g. Traditional or Local Ecological Knowledge, or Informal Traditional Knowledge). Despite memory loss inherent with this type of data, a well designed survey can provide invaluable data [5], [7], [8] for describing broad-scale trends and provide insight into patterns that may warrant further investigation. Often such studies describe important ecological patterns that would otherwise go unnoticed due to a paucity of data or insufficient timelines [5], [6], [30], [31] and are useful for generating new testable hypotheses and improving the knowledge base and compliance with management [6], [7]. Although these studies have traditionally used extractive resource users (i.e. fishers), recreational divers that regularly explore the marine realm over the course of years or decades and are familiar with local fauna, such as recreational dive instructors, may also provide vital information.

Here, we examine the value of recreational diver collected data for monitoring elasmobranch populations. First, we use a simulation program, *AnimDens*, to explore the density at which fish can expect to be detected by a roving diver and compare detection rates with the belt-transect and stationary point count techniques across a range of fish speeds, densities and survey-times. Then, for the purpose of using recreational divers to describe patterns in elasmobranch populations, we use a field survey to explore the effect of diver experience on detection and counts of the number of elasmobranchs present at a site. Finally, we use semi-structured interviews with experienced recreational dive instructors to explore the dive effort (number of dives) and spatial and temporal trends in shark populations in two regions of Thailand. Using this information, elasmobranch population descriptions using recreational diver observations could follow the lead of other citizen-based projects (e.g. eBird [3]) for a better understanding of broad spatial and temporal trends.

## Methods

### Ethics Statement

The nature of the work (interviews with divers) did not require any approval or permits regarding human or animal ethics.

### Comparing different UVC techniques

Scientists commonly utilize the belt-transect or stationary point count underwater visual census (UVC) techniques to count fishes in nearshore habitats. However, because of the sheer number of recreational divers worldwide it is likely that the roving technique can be used to gather more data than all other scientific UVC combined. To explore the value of the roving technique for detecting fish occurring at low density, we used a simulation approach to compare detection rates amongst these three UVC techniques across a range of fish densities and speeds.

**Figure 1:**
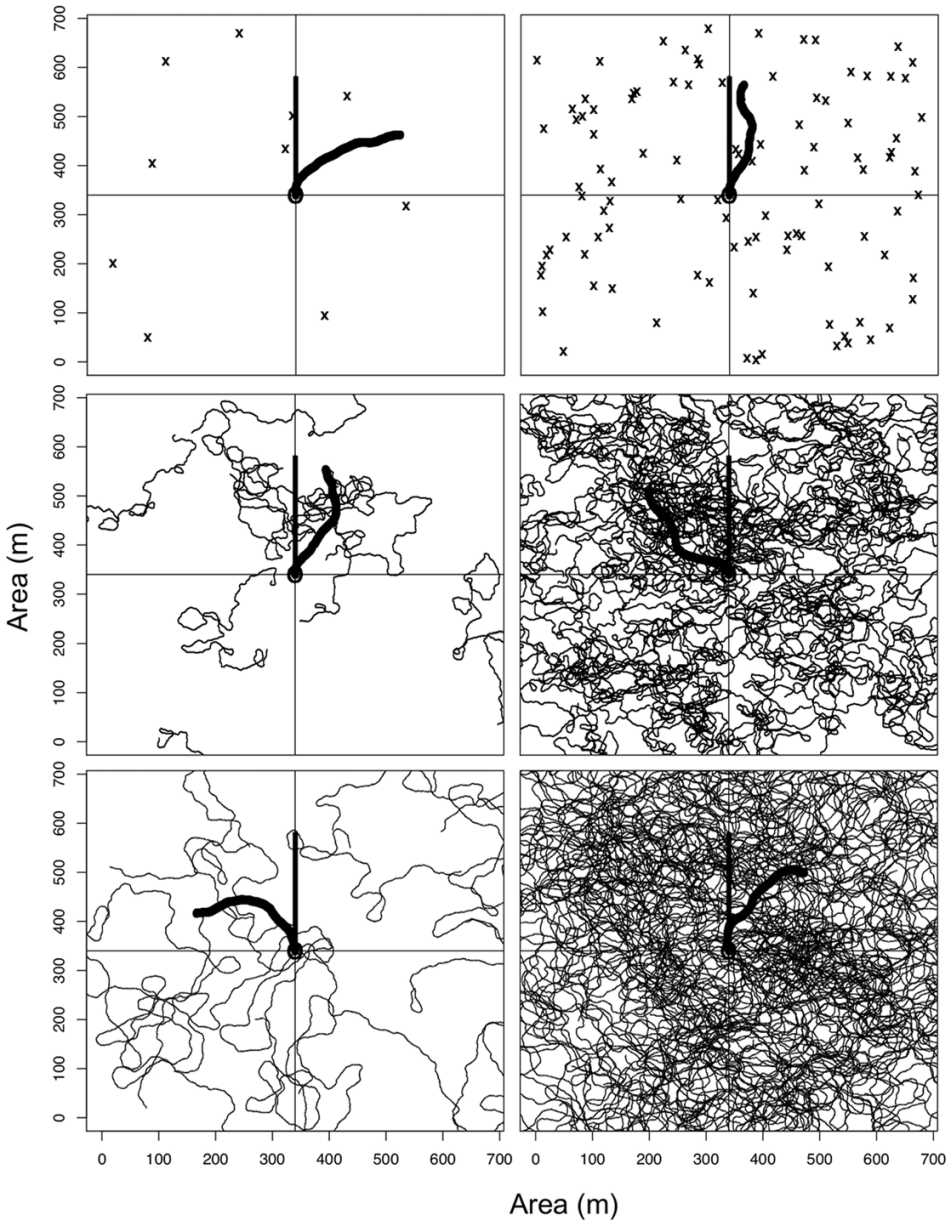
Example simulations showing the movement of fish with densities of 2×10^−5^ and 2×10^−4^ fish·m^−2^ (columns, from left to right) that moved at speeds of 0, 0.4 and 1.0 m·s^−1^ (rows, from top to bottom). Three divers were simulated, the stationary point count diver remained in the centre of the sampling area (circle), the belt-transect diver followed a straight path (bold straight line), and the roving diver followed a directed random path (bold curved line) over a 60 minute survey time. Belt-transect and roving divers travelled at 4 m·min^−1^. The area recorded by each diver is approximated by the length and width of the line that represents them.

The simulation program *AnimDens* was developed to explore the difference between observed counts and true densities made by belt-transect and stationary point count divers deploying non-instantaneous surveys under a range of sampling conditions [32]. Here, we adapted this simulation to include the roving technique using varied fish density and speed (Fig. 1; File S1). *AnimDens* provides a two-dimensional simulation of the visual census procedure representing both the movement of the divers using non-instantaneous sampling techniques and the fish at different densities and speeds.

For simplicity, the model assumed a sample area that was featureless, flat and 1 m deep. For each simulation, a diver from each of the three census methods was placed in the centre of the sample area with the same original orientation. The sample area was populated with fish that had a random distribution and random initial orientation. Although diver to shark interactions are considered to be an important factor in diver censuses, shark behaviour is certainly individual, location and species specific. The addition of this interaction would have required numerous assumptions and was not the main purpose of this study. For simplicity we therefore assumed no interaction.

In the stationary point count technique, surveyors remained still and recorded fish observed within a fixed distance [26]. In the belt-transect, divers swam along a straight line and recorded the animals they observed directly in front of them within a fixed distance of the line [20]. In the roving technique, the surveyor recorded the fish they observed, regardless of direction or distance as long as a reliable identification could be made, as they followed their regular dive activities [33]. Once the simulation started, the stationary point count diver remained still and the belt-transect diver moved straight forward at 4 m·min^−1^ [34]. The roving diver moved at 4 m·min^−1^ in a direction that changed within a random range of ±4°·2 s^−1^, which was based on observations of recreational divers in the field. At each time step the divers counted the fish they observed within the sample area. Although divers continuously count fish during field surveys, for time sake, we set the time step of each observation to 2 s.

Each run contained fish densities that ranged from 2×10^−6^ to 2×10^−1^ fish·m^−2^, which is approximately the maximum density reported for apex predators [21]. The fish were set to move at speeds of 0, 0.4, and 1.0 m·s^−1^, covering reasonable values attained by reef sharks [35]. Although there are anecdotes suggesting complex shark to diver interactions, for simplicity the direction of the fish was allowed to change within a random range of 45° left or right from the previous direction at each time step, which was based on personal observations of reef sharks (e.g., Caribbean reef shark *Carcharhinus perezi*, blacktip shark *C. limbatus* and blacktip reef shark *C. melanopterus*).

In each run, sharks and divers moved for 300 or 3600 seconds. The distance and angle between the three divers and each fish was calculated every two seconds to determine if the fish were within the field of view of the diver. For the stationary point count diver, all fish within 7.5 m and a field of view of 160° of the diver's orientation were detected. For the belt-transect diver, all fish directly in front of their position, within ±2 m of the transect line, to a distance equal to maximum visibility were recorded. For the roving diver, all fish within a distance of maximum visibility and a field of view of 160° of the diver's orientation were recorded. Note that fish that entered the survey area after the survey started were counted (i.e. non-instantaneous) and that the divers did not recount the fish they already recorded as they strive to do in the field [20], [36]. This simulation experiment was designed to compare the detection rates among the three different UVC methods for differing fish densities and speeds. Each model combination (Table 1) was run for 30 simulations. The means and standard errors are presented.

**Table 1:** Variable values used in the simulation *AnimDens*.

True density (fish⋅m^2^)	Fish speed (m⋅s^−1^)	Survey-time (s)	Visibility (m)	Transect-width (m)	Stationary radius (m)	Diver speed (m⋅min^−1^)
2.0×10^−6^	0	300	13	4	7.5	4
2.0×10^−5^	0.4	3600				
2.0×10^−4^	1.0					
2.0×10^−3^						
2.0×10^−2^						
2.0×10^−1^						

### Evaluating inexperienced versus experienced divers

Field studies were conducted to examine the influence of diver experience on the precision of detection and number of sharks or rays reported. Opportunistic surveys were carried out on tourist dive boats off the island of Koh Phi Phi, Thailand, in May 2008. Boats containing recreational and professional scuba divers were invited to participate. Dive teams consisted of at least one dive instructor and their clients who had a range of diving experience. On most occasions, several dive teams operated from the same boat and traveled in different directions. All divers were made aware of the project prior to the dive and asked to keep track of the number and species of sharks or rays they saw on each dive. Participants were instructed not to talk about their observations until the data was collected (File S2).

A total of 145 divers, 48 professional (e.g. dive instructors with >500 dives) and 97 recreational, with diving experience ranging from 2 to 5000 dives, participated in the field survey. These were grouped into inexperienced divers (≤20 dives: the number of dives required to begin a PADI Divemaster course; n = 28 divers) and experienced divers (>20 dives; n = 117 divers), with some divers being present on multiple dives. In teams of 2–9 (mean = 3.5), divers entered the water with an unknown number of sharks and rays, and were asked to conduct their normal dive activities, but to count the number of different sharks and rays they saw for each species. There were 1–12 different teams diving at the same time on a given dive (total number of dives = 7). Following the dive, participants were asked to report: 1) team number, 2) the number of dives they have done in their life, and 3) the number of sharks and rays they saw of each species on each dive.

Based on the collected data, we first evaluated whether inexperienced divers could detect the presence of sharks as well as experienced divers. To do this, we compared the presence or absence response of each shark or ray species for each diver to the response of their dive team (37 teams consisting of >2 divers) for dives where at least one shark or ray was reported (5 of all 7 dives). We assumed no false detections, where the report of the presence of a shark or ray was a correct response (e.g. they did not mistake another fish type for a shark or ray). Therefore, if a diver did not detect the presence of sharks or rays on a dive, but their dive team did, then the difference from the team for that diver would be one. However, if the diver and the team reported the same presence or absence, then the difference from the team would be zero. Diver experience (total number of dives in their life) was then compared to the difference between the diver and their team response. We also evaluated the variability of responses among inexperienced (≤20 dives) and experienced (>20 dives) divers using a chi-squared test.

Again, using teams with >2 divers and dives where at least one shark or ray was reported, we determined how much experience was required to precisely count the numbers of sharks or rays on a dive. Therefore, diver experience was compared to the difference between the number of sharks reported by each diver and the mean number of sharks reported by the dive team. We examined the variability of counts between inexperienced and experienced divers using the Bartlett's K-squared test of homogeneity of variance.

### Value of divers: effort, spatial and temporal patterns

The value of recreational divers was assessed using opportunistic semi-structured interviews with dive instructors in Thailand in May 2008 about their observations made during dives in the Andaman Sea (in the towns or islands of Phuket, Phi Phi, Koh Lanta and Krabi) and for the western Gulf of Thailand (on the islands Koh Tao, Koh Phangnan and Koh Samui). The value of the divers' observations was investigated in terms of sampling effort and descriptions of the spatial and temporal trends in elasmobranch populations. Divers were selected by snowball sampling (visiting dive shops and word of mouth). Participating divers must have had dive master or instructor training (from here on called "instructors"), led regular dive trips to sites in the surveyed region, have conducted at least 100 dives in their life, and have a minimum of 80 dives in the survey region. A minimum of 20 instructors were interviewed in each region (Andaman Sea and Gulf of Thailand) and attempts were made to locate at least three instructors per region that were diving in the 1990's or earlier. Instructors were asked their first name, dive shop affiliation, first and last year diving in the region, total number of dives in the region and in their life, and they were asked to list the dive sites visited most often. Most instructors had to make rough approximations of the number of dives done (e.g. number of dives per week x number of weeks per year x number of years). For each site listed, the instructors were asked how many times per week they visited that site on average, which shark species they encountered and the maximum number of each species seen on that particular site at one time (aka: "best day's catch" [5]). If the instructor had been visiting that site for more than one decade, they were asked for their maximum number of each species in each decade in a random sequence to prevent projection of their beliefs into the data (File S3).

Latitude and longitude of most of the listed sites were found online (e.g. www.wannadive.net), but were not available for sites that were listed as ‘secret’ (1 site) or visited by only few instructors (seven sites), which were sites for more specialized diving (e.g. wrecks and deep water). A few sites were so close to each other that instructors often regarded them as one site (e.g. Super Day includes King Cruiser, Anemone Reef and Shark Point). In cases where the individual sites were separated by the instructors, they were lumped into one site for the analyses. A few divers were unable to approximate the total number of dives they have done (in the region, in their life or on each site). In these cases the number was set to one, making effort (number of dives) a cautious minimum.

We investigated the value of recreational diver recollections in terms of their overall effort and their potential for describing spatial and temporal patterns of shark populations. For effort, we summarized the number of dives by instructor, region, site and number of sites visited. Then we used the observations to explore patterns in shark populations across sites, regions and decades in Thailand in terms of (i) presence/absence, (ii) average maximum school size (averaged across all divers that reported each species), and (iii) species diversity (total number of species reported). To explore the pattern of species accumulation with effort we modeled the number of shark species observed at a site as a function of the log of the number of dives per week using a generalized linear model (GLM) with a poisson error distribution and a log link. Since we would expect to observe zero shark species when effort is zero, we fit the model without an intercept term. Temporal trends were estimated for sites visited by the same divers in the 1990's and 2000's, and only included sites with more than two records for each decade. Dives per week was calculated as the sum of the number of dives done per week on a particular site.

## Results

### Comparing different UVC techniques

**Figure 2:**
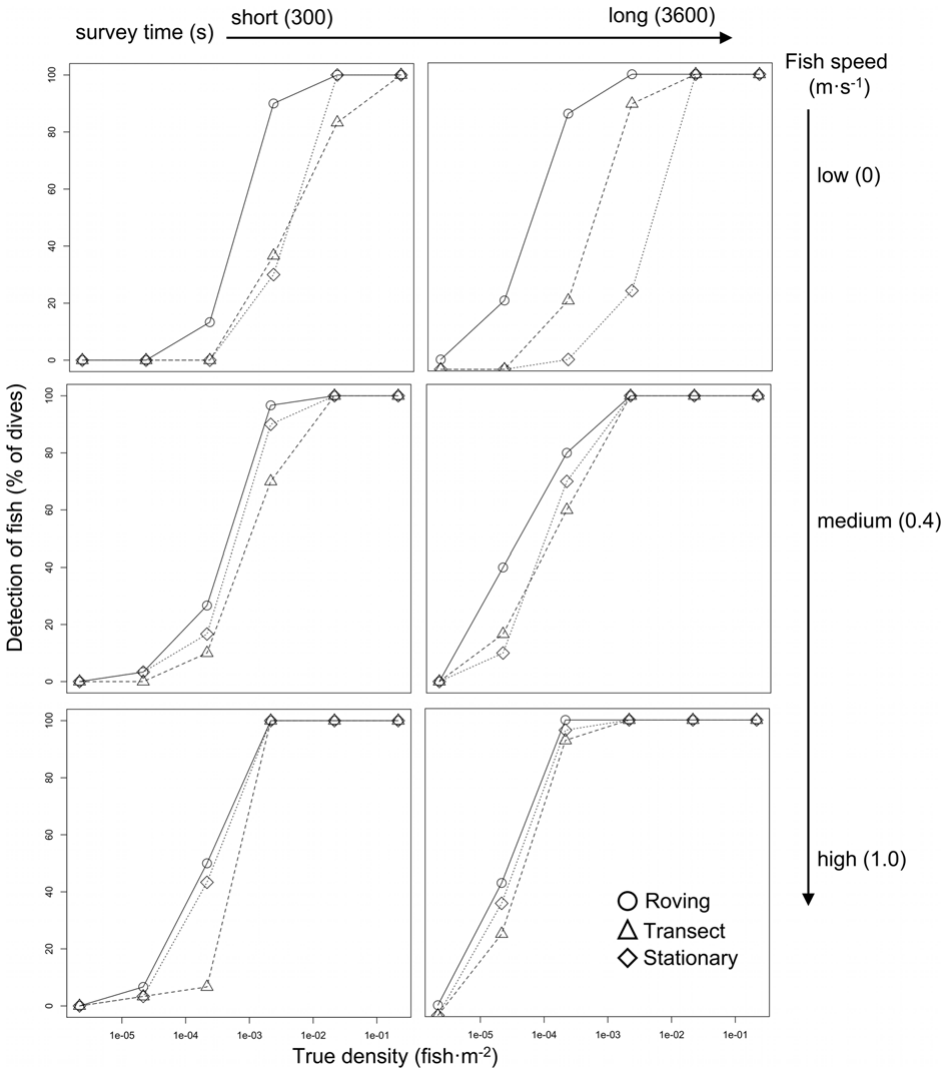
Percent of surveys (n = 30 simulations) where fish were detected across a range of fish densities (x-axis) for the roving diver (diamond, solid line), belt-transect (triangle, dotted line), and stationary point count (cross, dashed line). Columns (left to right) show 300 and 3600 second survey-times. Rows (top to bottom) show fish speeds of 0, 0.4, 1.0 m·s^−2^.

Over 30 simulations, the roving technique detected fish at lower densities than the belt-transect or the stationary point count techniques (Fig. 2); however, the difference was diminished with increased fish speed. As well, the roving technique detected fish more often at all fish speeds and densities, with the exception of the highest fish densities where all three UVC methods detected fish 100% of the time. For example, for 300 second survey-times, the roving technique started to detect stationary fish at densities one order of magnitude lower (i.e. 13% sighting frequency at a true density of 2×10^−4^ fish·m^−2^) than both the belt-transect and stationary point count techniques over 30 simulations (Fig. 2a). At fish speeds of 1.0 m·s^−1^, all three methods detected fish at a true density of 2×10^−5^ fish·m^−2^, the roving diver detected fish 7% of the time while the stationary or belt-transect divers detected fish 3% of the time (Fig. 2e). However, at higher densities, the effect of fish speed and survey-time was negligible and all three methods reliably detected the presence of fish in the survey area. Survey-time also affected the detectability of fish, with the effect being diminished with increased fish speed and density. For example, fish traveling at 0 m·s^−1^ were detected by all three methods at a true density of 2.0×10^−3^ fish·m^−2^, while they were detected by all three methods at 2.0×10^−4^ fish·m^−2^ for survey-times of 3600 s, one order of magnitude lower.

### Evaluating inexperienced versus experienced divers

**Figure 3:**
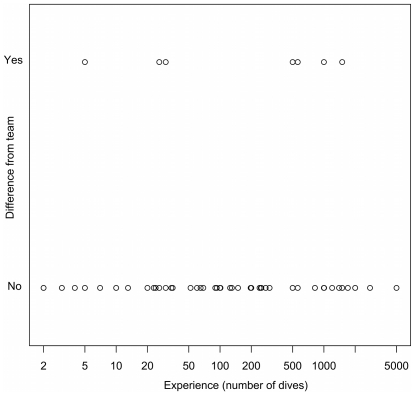
Comparison of participant diving experience and elasmobranch detection (presence or absence) with their respective team's detection, where sharks or rays were assumed to be present when at least one team member reported their occurrence (i.e. no false detections).

Participant diving experience did not affect the detection (i.e. presence) of sharks and rays on a dive (Fig. 3). Over 116 individual dives, seven divers differed from their team in terms of detection. The mean dive experience of these seven participants was 517 (± 215 SE) dives. Only one of these participants had <20 dives, two had 20–30 dives, and the other four had ≥500 dives, arguably experienced divers. The overall variability between inexperienced and experienced divers was not significantly different (Chi-squared p = 0.86).

**Figure 4:**
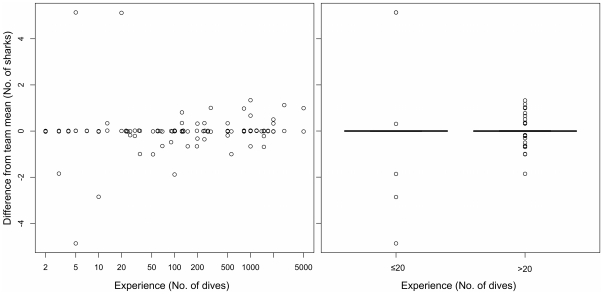
Comparison of a) participant diving experience and the difference between the number of elasmobranchs reported by the individual and their team mean, and b) the variability of counts for inexperienced (≤20 dives) and experienced (>20 dives) divers.

Inexperienced divers also reported similar counts of sharks and rays compared to experienced divers (Fig. 4). The variability amongst the most experienced divers (≥1000 dives) was ≤1.3 elasmobranchs (Fig. 4a). All outliers that were more than two times this value (>2.6 sharks) occurred for divers with ≤20 dives (n = 4). Although the overall outliers were greater for the inexperienced divers (differing by up to 5 elasmobranchs; Bartlett's K-squared, p<0.0001), the means and variance were not significantly different (t-test, p = 0.89) (Fig. 4b).

### Value of divers: effort, spatial and temporal patterns

In total, 49 instructors contributed their dive observations, 29 from the Gulf of Thailand and 20 from the Andaman Sea. Combined, these divers have done ∼83,982 dives in Thailand, 60,841 in the Gulf of Thailand (average 2,097±333 SE) and 23,141 in the Andaman Sea (average 1,157±229 SE). On average, 65% of all dives performed by a diver (i.e. their experience) are in these respective regions. Divers regularly visited 19 and 10 sites in the Gulf of Thailand and Andaman Sea, respectively. Site visits, by all divers combined, ranged from 1 to 156 dives per week (total = 743, average = 27±7 SE). Because some of these divers were not diving for the entire decade at this rate there is a large discrepancy between the total number of dives per site when extrapolated to the entire decade (385,840 dives) and the total number of dives in the area (83,982).

**Figure 5:**
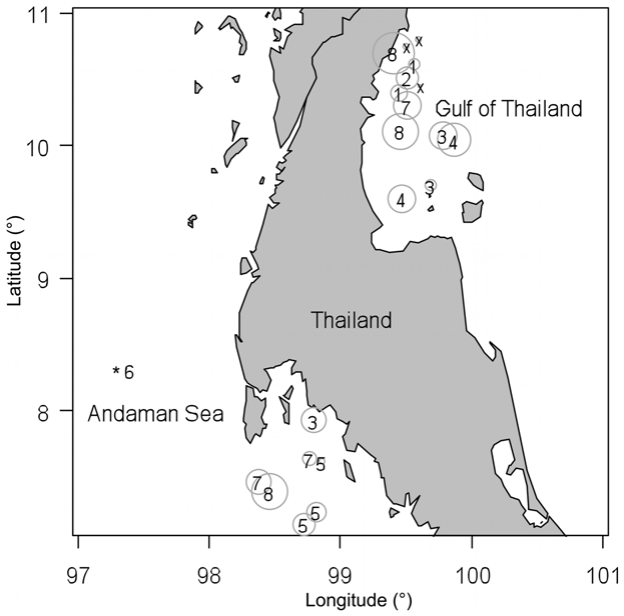
Map of the study area with sites regularly visited by dive instructors. Grey circles = effort in dives done weekly (range from 1 to 156 dives per week). Numbers = number of shark species observed across both decades on the site. X = sites where no sharks were observed. Note that 2 and 6 sites are not shown for the Andaman Sea and the Gulf of Thailand, respectively, because of unknown latitude and longitude values. No effort was given for the Similan Islands (*).

**Figure 6:**
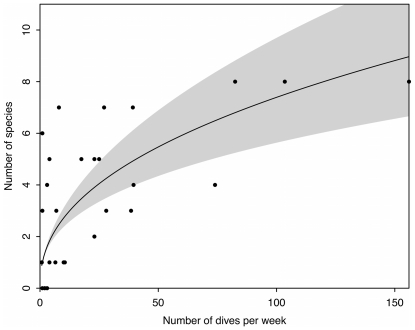
Comparison of effort (number of dives per week) and number of species observed for each site across all years. Trend was fitted using a generalized linear model with a poisson error distribution and a log link (null deviance: 183.8 on 29 degrees of freedom, residual deviance: 51.6 on 28 degrees of freedom).

Across both decades and all sites in Thailand, divers observed 10 shark species (Table 2)–many of which are unmistakable (e.g. whale–*Rhincodon typus*, leopard–*Stegostoma fasciatum*) and are known to occur in the region. Leopard (also called Zebra) sharks were observed on the highest number of sites (19 of 29 sites), followed by whale and blacktip reef sharks (16 each). School size ranged widely and blacktip reef sharks had the largest average school size (5.8±3.5 SE). Most species were observed in both study regions with the exception of bamboo (*Chiloscyllium* sp.) and oceanic whitetip sharks (*C. longimanus*) in the Gulf of Thailand and blacktip and nurse (*Nebrius ferrugineus*) sharks in the Andaman Sea. Site specific species richness ranged from 0 to 8 species (Fig. 5), with at least one shark species being observed on 82.7% of sites (5 sites did not have sharks reported). The number of species reported for a site increased with weekly dive effort (Fig. 6).

**Table 2:** Summary data for each region in Thailand for each shark species observed.

	Region	No. sites	Leopard	Whale	Blacktip reef	Whitetip reef	Blacktip	Nurse	Bamboo	Oceanic whitetip	Grey reef	Bull
No. sites	Andaman Sea	10	10	6	9	6	0	6	7	1	5	1
	Gulf of Thailand	19	9	10	7	5	3	3	0	0	5	3
Max. school size (SE)	Andaman Sea		4.1 (1.0)	1.3 (0.2)	4.3 (1.0)	1.9 (0.5)	0.0 (0.0)	1.3 (0.1)	2.8 (0.5)	1.0 (0.0)	5.7 (1.5)	1.0 (0.0)
	Gulf of Thailand		1.4 (0.1)	1.6 (0.2)	5.8 (3.5)	3.1 (1.5)	2.4 (0.9)	1.2 (0.2)	0.0 (0.0)	0.0 (0.0)	5.3 (1.7)	3.0 (1.1)

Shown are the number of sites visited where species were present and the average maximum school size for each species where they were observed with standard errors.

**Figure 7:**
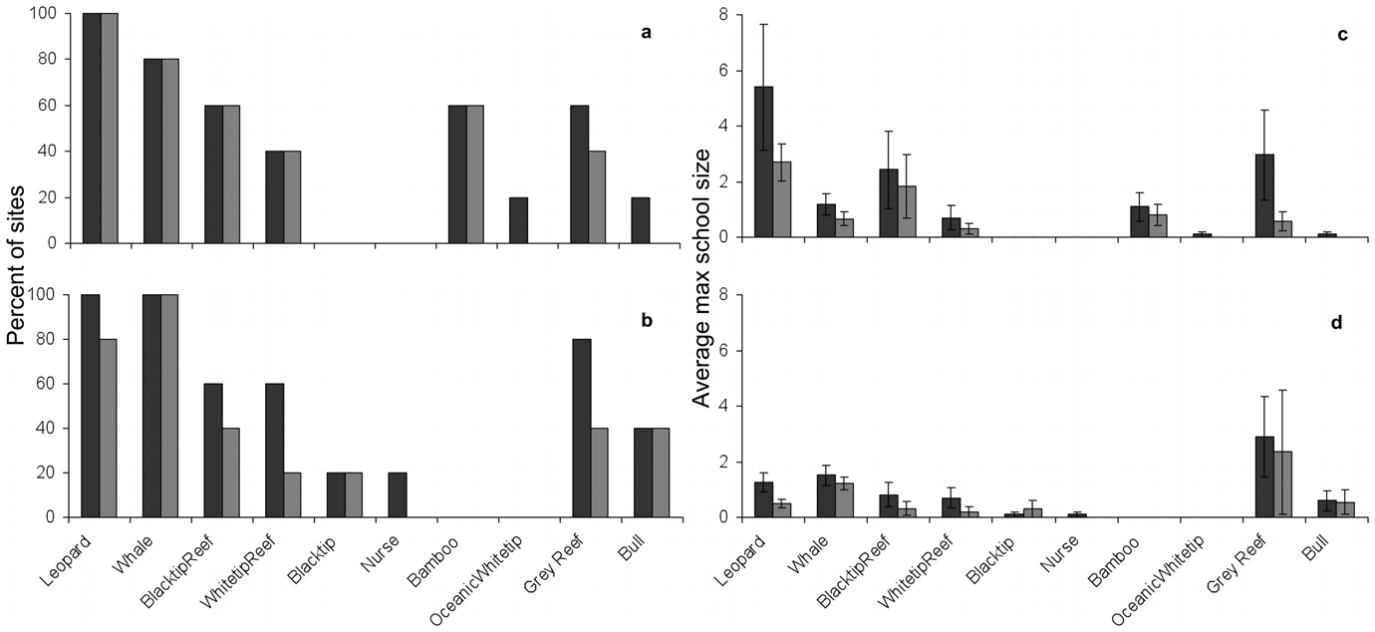
Temporal changes in species distribution (number of sites where species was observed, a and b) and average maximum school size (mean±SE, c and d) for 5 sites visited by divers in the 1990's (black bars) and 2000's (grey bars) for the Andaman Sea (a, c) and Gulf of Thailand (b, d). Note that only records made by the same divers in both decades were used.

Seven divers (three in Andaman Sea and four in Gulf of Thailand) provided observations for both the 1990's and 2000's at 10 sites (five in each region). Effort (number of dives per week) was constant on these sites between the two decades, with 23.5 and 42.5 dives per week in the Andaman Sea and Gulf of Thailand, respectively. There was little change in species distribution and maximum school size between the 1990's and 2000's (Fig. 7). In the Andaman Sea, three species were observed on one site fewer in the 2000's compared to the 1990's. Although seven out of eight species declined in maximum school size between the two decades, only leopard and grey reef (*Carcharhinus amblyrhynchos*) sharks had significant changes with the greatest decline (2.7 and 2.4 individuals, respectively). In the Gulf of Thailand, five species were observed on fewer sites in the 2000's compared to the 1990's, but only leopard sharks showed a significant decline in maximum school size. No species increased in distribution and only the blacktip in the Gulf of Thailand showed non-significant increase in maximum school size.

## Discussion

This study builds on our current knowledge of UVC for describing fish populations and demonstrates the value of using the broader diving community for censusing vulnerable and rare fish. Historically, sharks were abundant and widespread but many now occur at a fraction of their original abundance [16], [37] and may be threatened with extinction [38]. However, living sharks are increasingly valued in terms of their economic draw for tourism [39] and their important role in structuring marine ecosystems [37], [40]. In response, a range of management plans have been implemented to slow and ultimately reverse negative trends, from shark specific (e.g. anti-finning regulations and shark sanctuaries) to ecosystem based strategies (e.g. no-take marine reserves). Currently, there is limited infrastructure to monitor shark populations non-destructively, which is especially important for quantifying the success of different management and conservation measures where mortalities should be minimized. Therefore, our study provides important insight into the value of recreational divers for collecting data that may be useful for describing and monitoring broad-scale trends in elasmobranchs.

Under the conditions of our simulation, the roving diver technique, which is commonly used by recreational divers, was not inferior to the more scientific belt-transect and stationary point-count UVC techniques. Our simulation results show that the roving diver technique is the most adept for providing presence data on low density, rare and conspicuous fishes like elasmobranchs. This difference was largely a result of the roving diver covering more area during a survey, and the difference in detection rates between the three UVC techniques was reduced with increased fish density, fish speed and survey time–assuming that all methods counted fish they detected after the survey started (i.e. non-instantaneous). As well, because the roving diver technique censuses all fish from the beginning to the end of a dive, it would have the added benefit of capturing highly mobile species that may be wary or curious of divers [41]–[44], and seen at the beginning or end of a dive and would be missed by the other two UVC techniques that require an initial set-up period. Additionally, the two scientific UVC techniques do not commence until the diver is in place, usually near the bottom, therefore limiting searches to a fixed vertical distance from the bottom substrate, whereas the roving technique includes all species observed, regardless of their location in the water column. Therefore, the roving technique should be better suited for detecting species that occupy pelagic (e.g. blacktip shark) and surface (e.g. whale shark) waters.

There are two drawbacks of the roving diver technique compared to the belt-transect and stationary point count techniques. First, the roving technique does not record fish length, which thus excludes analyses of biomass. Addition of this measurement to the roving technique would require additional training [8] and is time consuming, thereby decreasing the time spent enumerating fish and would likely lower volunteer participation. The second drawback is that the roving technique does not delineate the area covered during a survey, which is essential for estimating density. However, if effort (visibility and bottom time) and environmental characteristics (habitat type, depth, date) are recorded for each dive, the data may be standardized and relative changes through space and time determined using appropriate modeling techniques [15].

In addition to comparing these three censusing techniques, our simulation results may provide insight into the true density of a population based on detection rates (i.e. presence/absence rate) for a given survey type. For example, if a study utilizing 4 m wide belt-transects for 5 min traveling at 4 m·min^−1^ detected the presence of a stationary animal on 40% of its surveys, then the true density of that animal would be approximately 1.0×10^−3^ individuals·m^−2^. However, for animals moving at 1.0 m·s^−1^ under the same sampling scenario as above, the true density would be closer to 1.0×10^−4^ individuals·m^−2^, or one order of magnitude smaller. Obtaining approximate density estimates this way could be very useful for rare species, like sharks, that are often disregarded because individuals rarely enter survey boundaries.

Divers with a wide-range of skill levels have the potential to provide important data on elasmobranchs that may be used in distribution and population abundance monitoring. In our field studies in Thailand, we found that inexperienced divers (≤20 dives in their life) detected the presence of elasmobranchs as well as experienced divers. This is important because occurrence data alone can provide valuable information that can be used to monitor broad-scale trends in abundance, distribution and diversity [45]–[47]. Our results also indicate that counts of elasmobranchs obtained from inexperienced divers are precise compared to experienced divers. Although the absolute value of the outliers was greater for inexperienced divers, the variance was smaller and inexperienced divers were just as likely to underestimate abundance as they were to overestimate abundance. This suggests that, if the observations of multiple divers are combined, inexperienced divers should be able to provide useful data.

The value of recreational divers for describing trends in shark populations lies in their ongoing observational effort (i.e. number of dives) on a large number of sites around the world. High effort is important for detecting rare species and the presence or absence alone can provide insight into the distribution and relative abundance of elasmobranch populations [16], [21], [22], [24] or ecosystem health as a whole [48]. Using the observations of 49 dive instructors, conducting more than 83,000 dives in the Andaman Sea and Gulf of Thailand we were able to provide some new quantitative descriptions on the spatial and temporal trends of 10 shark species, all of which have vulnerable or near threatened status and have either globally declining or unknown population trends [49].

Because Thailand has a high human population, substantial habitat destruction, strong fishing pressure that has persisted for decades and very limited management initiatives [50] we would expect sharks to be absent or at such low abundance that they would not be detected by divers similar to other populated regions of the world [16], [21]–[23]. However, this was not the case. All interviewed divers observed sharks in the study region and most sites (>80%) had at least one species and four sites had six species. (Note that attractants (e.g. chum or bait) were not used to lure sharks to divers in this region.) Although we did not collect data on the regularity of seeing sharks, all the recreational dive instructors that were interviewed asserted that they saw at least one shark (usually leopard, blacktip reef, whale or bull) on a fairly regular or seasonal basis. For example, on one site in the Gulf of Thailand, up to eight bull sharks were seen on a daily basis for ∼8 months every year. This particular site likely has >300 divers per day and the sharks are not artificially attracted in any way.

The reported declines between the 1990's and 2000's were relatively small for maximum school size (maximum decrease = 2.7 individuals) and number of occurrence sites (maximum decrease = 2 sites). However, since overfishing and habitat loss is high in Thai waters, causing the decline of many shark species including those observed by divers in the current study [50], it is likely that contemporary populations are a small fraction of their historical abundance and the changes between the two recent decades are inconsequential compared to longer term changes. For example, leopard, whale, nurse and most (17 of 29) requiem sharks (Carcharhinidae) are declining or disappearing in the study region and were historically more abundant than they are today [49], [51]. Although the extent of these declines is not shown in our trend analysis they are consistent with the overall rarity and small school sizes of these species in our data. Additional evidence of the magnitude of loss may be provided by our findings from the dive site "Shark Island" in the Gulf of Thailand. Surely, this site was not named for the occasional couple of sharks that are observed today. We could find only one dive instructor to provide observations for the 1970's. However, based on one, three and 20 divers' observations from the 1970s, 1990s, and 2000s, respectively, the maximum number of blacktip reef sharks went from 30 to 3 to 2 and whitetip reef sharks went from 10 to 2 to 0 in each decade–both declining by one order of magnitude from the 1970's to the 1990's. These findings indicate that more longer term historical data may be needed to understand how populations have changed through time in areas with a long history of exploitation.

Our study suggests that observations made by recreational divers show promise for divulging important trend information for conspicuous species, like elasmobranchs. However, as we did not perform trials of identification, although dive operators have been accurate at identifying common species [52] and all participants in the field study reported the same species as their respective team, mistakes in species identification should be considered when interpreting observational data. Analysis of the known minimum depth of all elasmobranchs puts 187 sharks and 216 rays and skates in the world within a reasonable maximum depth range obtained by recreational divers (set to 35 m). Although many of these are unmistakable (e.g. whale shark) or are too rare to be seen by a diver (e.g. Irrawaddy river shark, *Glyphis siamensis*), others occupy the same niche and have similar morphologies (e.g. blacktip and spinner, *C. brevipinna*), making accurate identifications challenging. Identification is likely improved with reduced distance and increased frequency and duration of encounters and with quality photographs. Similarly, accuracy would be diminished for short, distant and rare encounters–factors that would be expected to affect recreational and scientific divers alike. For example, in the current study, bull and grey reef sharks have overlapping niches and similar morphologies and may be misidentified by untrained observers. Therefore, caution may be needed when interpreting changes in distribution or abundance of these species; however, in this case photographs were used to verify the presence of both species and both showed similar trends which indicates a true decline.

Although there is no replacement for the data provided by expert scientific observers, we suggest that recreational divers, reporting their observations from daily dive activities, could provide invaluable broad-scale and long-term information that would allow for early identification of changes in elasmobranch populations. Therefore, an important next step may be to follow the lead of other citizen science projects (e.g. eBird [3]) to encourage divers to participate in elasmobranch research, to gather useful data, and to analyze the data appropriately. Harnessing the high effort of recreational divers in a standardized way could provide useful population information across a range of spatial and temporal scales. These data may be used to define contemporary baselines against which future changes may be measured, designate priority conservation areas, compare current observations with historical anecdotes to understand population changes through time, and measure the relative success of different management strategies for protecting elasmobranchs.

## Supporting Information

File S1 **Code for the simulation AnimDens_B (written in R).**
(R) Click here for additional data file.

File S2 **Observations and experience records from recreational divers in Thailand.**
(CSV)

File S3 **Recollections of observations and experience from dive instructors in Thailand.**
(CSV)
